# Intimate partner violence and eating disorders among married women: the mediating role of emotion dysregulation

**DOI:** 10.3389/fsoc.2026.1747374

**Published:** 2026-05-22

**Authors:** Rula Odeh Alsawalqa, Nisreen Mahmod Alkaraki, Lubna Aladaileh, Ann Mousa Alnajdawi, Roqaya Alreesi

**Affiliations:** 1 Department of Sociology, University of Khorfakkan, Sharjah, United Arab Emirates; 2 Department of Sociology, The University of Jordan, Aljubeiha, Jordan; 3 Department of Social Work, Al-Balqa Applied University, As-Salt, Jordan

**Keywords:** eating disorder, emotion regulation, intimate partner violence, Jordan, women

## Abstract

This longitudinal study investigated the relationships between exposure to intimate partner violence (IPV), difficulties in emotion regulation (DERS), and eating disorder symptoms (ED) among married women in Jordan. Results showed strong positive correlations between all forms of IPV—including economic control, economic exploitation, psychological abuse, physical violence, emotional abuse, and harassment—and all dimensions of DERS, including nonacceptance of emotions, impulse control difficulties, and limited access to emotion regulation strategies. DERS were also significantly associated with ED symptoms, such as dieting behaviors, binge eating and food preoccupation, and oral control. Longitudinal analyses revealed that ongoing exposure to IPV led to significant increases in DERS and ED symptoms over 6 months, particularly in impulse control difficulties and limited access to regulation strategies. Changes in DERS significantly predicted corresponding changes in ED symptoms, even after controlling for baseline levels and demographic variables. Mediation analyses confirmed that DERS partially mediated the relationship between IPV and ED symptoms, highlighting the critical role of emotion regulation in this pathway. Furthermore, women exposed to multiple types of IPV exhibited significantly higher DERS and ED symptom scores compared to those exposed to a single type of violence. Structural equation modeling supported these findings, showing that exposure to IPV increases DERS, which in turn exacerbates ED symptom severity, while a partial direct effect of IPV on ED symptoms remains. These results underscore the compounded negative impact of IPV on emotional regulation and eating pathology among married women, emphasizing the importance of interventions targeting emotion regulation to mitigate the effects of IPV on disordered eating.

## Introduction

Psychological well-being is influenced by a complex interplay of emotional, behavioral, and interpersonal factors. Among these, Emotion Dysregulation (DERS), experiences of violence, and eating disorders (ED) have each been identified as significant concerns due to their adverse effects on individuals’ mental and physical health. Increasing evidence suggests that these phenomena often co-occur, creating compounded risks and challenges for those affected (Holmes et al., 2023; Huston et al., 2019). Emotion Dysregulation (DERS) refers to deficits in the ability to be aware of, understand, and accept emotions; the inability to control impulsive behaviors and act in accordance with desired goals when experiencing negative emotions; and difficulty in accessing effective strategies for regulating emotions (Gratz and Roemer, 2004). Effective emotion regulation is essential for success, enhancing self-esteem and problem-solving abilities. Difficulties in regulating emotions can lead to dissatisfaction with oneself, impaired decision-making, and increase the risk of emotional disorders such as anger, depression, anxiety, and mood instability (Cengiz and Gürel, 2020; Gross, 2015). Psychologically, poor emotion regulation is linked to mental health problems, while physically, chronic emotion dysregulation contributes to stress-related illnesses including hypertension, cardiovascular issues, and weakened immune function (John and Gross, 2004; Cengiz and Gürel, 2020). Behaviorally, these difficulties may result in maladaptive behaviors like substance abuse, aggression, and social withdrawal (Aldao et al., 2010).

Research shows that dysregulated negative emotions and marital satisfaction or discord are positively associated with Intimate Partner Violence (IPV) perpetration (Bliton et al., 2016; Stith et al., 2008; Birkley and Eckhardt, 2015). IPV is abuse or aggression by a current or former spouse or dating partner, ranging from a single incident to ongoing, severe episodes over time. It includes physical violence (using force to harm), sexual violence (forcing unwanted sexual acts or contact), stalking (repeated unwanted attention causing fear), and psychological aggression (verbal or non-verbal actions aimed at harming or controlling a partner) (Centers for Disease Control and Prevention, 2024).

Birkley and Eckhardt (2015) found that IPV perpetration was moderately correlated with anger, hostility, and internalizing negative emotions. This relationship was stronger among individuals who engaged in moderate to severe IPV compared to those involved in low to moderate IPV, and it remained consistent across perpetrator sex, measurement methods, relationship types, and populations. Similarly, Shorey et al. (2011) demonstrated that both broad DERS and specific emotion regulation problems were associated with dating violence perpetration, effectively distinguishing between individuals who had and had not perpetrated aggression. In the same vein, Maloney et al. (2023) reported a small to moderate association between emotion dysregulation and physical, psychological, and sexual IPV perpetration, noting that the strength of this relationship varied depending on the specific emotion dysregulation constructs examined, whether emotion regulation predicted increases or decreases in IPV perpetration, and the type of IPV assessed.

Berke et al. (2016) further examined the role of masculine discrepancy stress, finding that men experiencing this stress were more likely to use physical violence against their partners due to emotion dysregulation. Specifically, discrepancy stress led to problems in managing emotions, which in turn resulted in physical violence. However, in the case of sexual violence, emotion dysregulation only partially mediates the relationship between discrepancy stress and sexual violence. Longitudinal evidence from Simpson et al. (2025) revealed that, at the within-person level, increases in cumulative IPV among young women over a 28-month period were associated with increases in emotion dysregulation. Specifically, the accumulation of IPV experiences over time corresponded with greater difficulties in controlling impulsive behaviors when distressed and in accessing effective emotion regulation strategies. At the between-person level, higher average cumulative IPV was linked to elevated mean levels of global emotion dysregulation, including four specific dimensions: difficulties accepting emotional responses, challenges in accessing emotion regulation strategies, struggles engaging in goal-directed behaviors, and problems controlling impulsive behaviors when distressed.

Moreover, various forms of IPV—including psychological aggression, sexual coercion, physical assault, and injury—have been shown to negatively affect victims’ emotion regulation abilities, which in turn exacerbate trauma symptoms (Taccini et al., 2024). Kim and Capaldi (2004) also identified significant associations between the psychological and behavioral characteristics of romantic partners. Their findings indicated that women’s antisocial behavior and depressive symptoms were significantly related to men’s physical and psychological aggression, both concurrently and longitudinally, whereas men’s risk factors had limited influence on their own aggression toward partners. Although this study did not directly examine DERS, existing literature links psychological traits such as depression and antisocial behavior to emotion regulation problems. These findings underscore the importance of considering complex psychological factors in understanding IPV, aligning with the current study’s hypotheses regarding the interplay between exposure to IPV, emotion dysregulation, and ED symptoms.

Eating disorders (ED) are complex and potentially life-threatening conditions, associated with elevated mortality rates due to medical complications and suicide. They often co-occur with psychiatric and medical comorbidities, including anxiety, mood disorders, and neuroendocrine or gastrointestinal complications, which can exacerbate symptom severity and negatively impact treatment outcomes (Hambleton et al., 2022). ED also represents a form of control that individuals exert over their bodies, particularly in response to the control exercised by abusive partners. Exposure to physical, psychological, and sexual violence is linked to disorders such as anorexia nervosa, bulimia nervosa, and binge eating disorder. Gender differences have been observed in the relationship between sexual violence and ED symptoms, highlighting the nuanced ways in which abuse influences eating behaviors (Bretaña et al., 2025).

In a network meta-analysis study, Leppanen et al. (2022) found that rumination and non-acceptance of emotions are the emotion regulation strategies most strongly associated with ED symptoms, whereas difficulties in cognitive reappraisal show the weakest association. Rumination—whether general or illness-specific, such as preoccupation with food, weight, and shape—is prominent in acute stages and interacts with negative mood, sustaining symptoms. Non-acceptance of emotions leads to reduced use of adaptive strategies and increased reliance on maladaptive ones, like suppression and avoidance, reinforcing rumination and negative mood. This association is weaker among individuals with low Body Mass Index (BMI), possibly because starvation itself acts as an emotion regulation strategy by dampening physiological and emotional responses.

Emotion Dysregulation (DERS) increases susceptibility to ED symptoms, including loss-of-control eating and emotional eating. These difficulties are more strongly linked to ED in females than males, while BMI does not significantly affect this relationship (Zhou et al., 2025). Brockmeyer et al. (2014) further confirmed that all ED types are generally associated with DERS, except binge-eating disorder (BED), which shows fewer problems in certain emotion regulation areas. Specific difficulties, such as impulse control, may be more pronounced in subtypes like the binge/purge form of anorexia nervosa. Behavioral traits like inefficacy, fear of maturity, and impulsivity are strongly associated with fear of loneliness among those with ED. Although fear of loneliness does not directly affect experiences of IPV, it moderately impacts physical, psychological, and social violence depending on levels of social withdrawal (Momeñe et al., 2022). Momeñe et al. also highlighted that individuals with ED are more likely to experience IPV, with a high prevalence of such violence in this group. They noted a bidirectional relationship between ED and IPV, where each can influence the other, though the exact mechanisms remain unclear. Social isolation and fear of loneliness may contribute to sustaining these violent relationships. Moreover, significant associations have been identified between eating disorders, lifetime intimate partner violence, depression, and post-traumatic stress disorder (PTSD) (Huston et al., 2019).

In Jordan, women face significant emotion dysregulation, including difficulties in clearly expressing, attending to, accurately identifying, and valuing their emotions. They also struggle to maintain focus on tasks and to find effective ways to improve their mood during distress, often experiencing overwhelming emotions (Abdulhamid, 2023). Working married women additionally suffer from marital and emotional burnout, which has harmful health effects such as headaches, ED, irregular heart rate, and stomach pain (Alsawalqa, 2017; Tahat, 2024). They also experience multiple forms of IPV, including psychological, physical, emotional, economic abuse, and neglect (Alsawalqa R., 2021).

Most Jordanian women, situated within strict patriarchal structures that increase their vulnerability to IPV, resist violence through daily covert actions aimed at avoiding immediate harms like beatings, divorce, family breakdown, and financial instability. Bound by social, financial, and familial pressures, and fearing the consequences of divorce, many remain with abusive husbands. Common resistance strategies include silence, behavior changes, avoidance, isolation, patience, taking responsibility, attempting to change the partner’s behavior, bargaining, refusing routine household chores, yelling, blaming, and seeking social support. These resistance efforts often result in reduced or ceased physical violence, divorce, decreased social support, social stigma, marital and emotional burnout, financial difficulties, and sometimes increased violence. Furthermore, these women frequently suffer negative psychological effects, including depression, stress, anxiety, loss of self-esteem, eating and sleeping disorders, and isolation (Alsawalqa et al., 2022; Sa’Deh et al., 2024). Despite the growing body of literature on DERS, ED, and IPV, little is known about how these factors interact, particularly in terms of potential mediating mechanisms. This gap is especially evident among married women in Arab Islamic societies, where sociocultural norms, gender roles, and patriarchal structures may uniquely shape these relationships. Therefore, the present study aims to investigate the interplay between emotion dysregulation, IPV, and ED symptoms among married Jordanian women, with special attention to the potential mediating pathways linking these variables.

### Research hypotheses

*H1*: There is a statistically significant positive association between exposure to IPV and DERS.

*H2*: DERS are positively and significantly correlated with symptoms of ED.

*H3*: IPV is significantly positively correlated with the manifestation of ED symptoms.

*H4*: DERS significantly increase over a six-month period among women exposed to IPV.

*H5*: ED symptoms significantly increase over six months among women experiencing DERS.

*H6*: Changes in DERS over time predict changes in ED symptoms among women exposed to IPV.

*H7*: DERS mediates the relationship between exposure to IPV and the emergence of ED symptoms among women.

*H8*: Women exposed to multiple types of IPV concurrently exhibit higher levels of DERS and ED symptoms compared to women exposed to a single type of IPV.

## Methods

### Participant recruitment and data collection

A purposive sample of 308 married working women aged 27 to 51 from Amman was selected. Most participants hold a bachelor’s degree (71.4%), with around half employed in the public sector. Nearly 88% have children aged between 2 and 6 years. Household monthly income for 77.9% of families ranged from 610 to 3,800 Jordanian dinars. Most spouses are employed (87.7%), with 50% holding bachelor’s degrees. Approximately 70% of participants live with their spouses in rented homes. Recruitment was facilitated by the researchers’ prior experience in studies on violence against women in Jordan, particularly among married women, which helped establish trust for this longitudinal study aiming to explore psychological and behavioral changes over time.

The longitudinal design enables observation of changes and causal relationships within the same cohort over time without variable manipulation. The study commenced in November 2024, with initial assessments followed by periodic contact via phone calls, WhatsApp messages, and home visits to minimize attrition. Follow-up assessments were conducted 6 months later in May 2025.

The initial target sample size was 510; however, due to challenging personal circumstances, including divorce and emigration, many participants withdrew. Notably, 77.9% of those remaining reported living in abusive relationships but felt unable to leave due to concerns for their children and social stigma. Some also indicated familial acceptance of marital abuse as normative and endorsed traditional patriarchal authority. Ethical approval for this study was granted by (Blinded for review), Ref: 8/2025.

### Measures

Several measures were used in this study to assess economic abuse, psychological abuse, difficulties in emotion regulation, and risk for eating disorders among Arab women. The Eating Attitudes Test-26 has been translated and validated in Arab populations, including Saudi Arabia and Lebanon, demonstrating good internal consistency and construct validity, with minor adaptations to reflect cultural nuances in eating attitudes (Haddad et al., 2020). The Difficulties in Emotion Regulation Scale – Short Form (DERS-SF) has also been translated and psychometrically evaluated in Arab contexts, particularly in Jordan, showing acceptable reliability and validity (Fekih-Romdhane et al., 2023; Abdulhamid, 2023). The Community Composite Abuse Scale was carefully translated into Arabic using forward-back translation, expert review, and focus groups to ensure conceptual and linguistic equivalence, and the Arabic version has demonstrated acceptable reliability and validity in detecting multiple forms of intimate partner abuse (Alhabib et al., 2013). It was also applied in Jordan by Alsawalqa R. (2021) to measure physical abuse, emotional abuse, and harassment among women in both rural and urban settings, confirming its applicability in an Arab research context. The Scale of Economic Abuse (SEA) has been examined in Jordan, where it was used to assess economic abuse among married women (Alsawalqa R., 2021), and research in Lebanon has also documented experiences and perceptions of economic abuse within the population (Usta et al., 2013). While the Profile of Psychological Abuse of Women is widely used internationally, there is limited published evidence of its direct application or formal cultural adaptation in Arab contexts. Overall, these measures provide robust assessment, but future research should continue to culturally adapt and validate instruments to ensure accurate evaluation of intimate partner violence, difficulties in emotion regulation, and risk for eating disorders in Arab populations.

1. The Scale of Economic Abuse (SEA): developed by Adams et al. (2008), consists of 28 items measuring two main types of economic abuse within intimate relationships: economic control and economic exploitation.
2. Profile of Psychological Abuse of Women: a 21-item scale that measures a wide variety of psychological aspects (Sackett and Saunders, 1999).
3. The Community Composite Abuse Scale: a 28-item scale that measures physical abuse (10 items), emotional abuse (14 items), and harassment (4 items) (Loxton et al., 2013).
4. The Difficulties in Emotion Regulation Scale - Short Form (DERS-SF; Kaufman et al., 2015) is an 18-item measure assessing emotion regulation difficulties in adults across six subscales: nonacceptance of emotional responses, difficulties engaging in goal-directed behavior, impulse control problems, lack of emotional awareness, limited access to regulation strategies, and lack of emotional clarity. Items are rated on a 5-point Likert scale from 1 (Almost Never) to 5 (Almost Always), with higher scores indicating greater difficulties. This scale captures the frequency and intensity of challenges in regulating emotions.
5. The Eating Attitudes Test-26 (EAT-26), developed by Garner et al. (1982), is a 26-item self-report measure assessing risk for eating disorders through attitudes, feelings, and behaviors related to eating. It includes three subscales: Dieting, Bulimia and Food Preoccupation, and Oral Control. Responses are given on a six-point Likert scale from “Never” to “Always,” with the top three frequencies scored from 1 to 3 and the lower three scored as 0. Higher total scores indicate greater risk of disordered eating. Some items are reverse scored for accuracy.

### Statistical analysis

Data analysis was conducted using IBM SPSS Statistics (Version 25). Descriptive statistics, including means and standard deviations, were computed to summarize sample characteristics and study variables. The reliability of measurement instruments was assessed using Cronbach’s alpha coefficients to ensure internal consistency. Data normality was evaluated using the Kolmogorov–Smirnov and Shapiro–Wilk tests, supported by skewness and kurtosis values within acceptable ranges. Pearson correlation coefficients were calculated to examine bivariate relationships among key variables. Longitudinal changes in DERS and ED symptoms were analyzed using paired-sample t-tests and repeated measures ANOVA. Group differences based on types of IPV exposure were tested with independent samples t-tests. Hierarchical regression analyses assessed predictive relationships over time, while mediation analysis was performed using the PROCESS macro with bootstrapping to explore indirect effects. A significance level of *p* < 0.05 was applied throughout all analyses.

Furthermore, Structural Equation Modeling (SEM) was employed to test the hypothesized relationships among IPV exposure, DERS and ED symptoms, including the mediating role of DERS. SEM analyses were conducted using IBM AMOS (Version 26). Prior to testing the full structural model, confirmatory factor analyses (CFAs) were performed to validate the measurement models for each latent construct: IPV, DERS, and ED Symptoms (EAT-26). Model fit was assessed using multiple indices, including the Comparative Fit Index (CFI), Tucker-Lewis Index (TLI), Root Mean Square Error of Approximation (RMSEA), and Standardized Root Mean Square Residual (SRMR). Acceptable model fit criteria were defined as CFI and TLI values above 0.90, RMSEA below 0.08, and SRMR below 0.08. The hypothesized structural model examined direct and indirect effects among the latent variables. Bootstrapping with 5,000 resamples was used to test the significance of indirect (mediated) effects, with 95% bias-corrected confidence intervals. All variables were treated as continuous, and maximum likelihood estimation was employed. Model parameters were interpreted using standardized path coefficients (*β*), standard errors, and *p*-values, with a significance threshold set at *p* < 0.05.

## Results

### Descriptive statistics of main study variables

The descriptive statistics revealed moderate levels across all measured domains among the women in the sample. Within the violent variables, emotional abuse showed the highest mean score at 3.10 (SD = 0.85), followed by economic control (*M* = 2.90, SD = 0.80), economic exploitation (*M* = 2.80, SD = 0.72), harassment (*M* = 2.45, SD = 0.95), and physical abuse (*M* = 2.30, SD = 0.90), respectively. This pattern indicates that emotional abuse was the most frequently experienced form of IPV, while physical abuse was the least reported. Regarding ED symptoms, dieting was the most prevalent dimension among the participants, with a mean of 2.60 (SD = 0.70), followed by oral control (*M* = 2.50, SD = 0.65) and bulimia and food preoccupation (*M* = 2.40, SD = 0.68). For DERS, the highest means was observed in impulse control difficulties (*M* = 2.90, SD = 0.75), indicating significant challenges in managing impulsive behaviors. This was followed by nonacceptance of emotional responses (*M* = 2.75, SD = 0.78), limited access to emotion regulation strategies (*M* = 2.70, SD = 0.70), difficulty engaging in goal-directed behavior (*M* = 2.65, SD = 0.80), lack of emotional clarity (*M* = 2.60, SD = 0.74), and lack of emotional awareness (*M* = 2.55, SD = 0.82), respectively.

### Assessment of reliability, validity, and data normality

The reliability of the measurement instruments was assessed using Cronbach’s alpha coefficients, yielding high internal consistency across all scales: 0.89 for the DERS, 0.92 for the IPV scale, and 0.87 for the Eating Attitudes Test-26 (EAT-26). Construct validity was confirmed through both exploratory and confirmatory factor analyses, with model fit indices meeting accepted thresholds (CFI > 0.90, TLI > 0.90, RMSEA < 0.08).

Tests of normality, including the Kolmogorov–Smirnov and Shapiro–Wilk tests, indicated that the data approximated a normal distribution (*p* > 0.05), supported by skewness and kurtosis values within the acceptable range of ±1. The sample size of 308 married women was deemed sufficient for advanced statistical analyses such as regression and structural equation modeling (SEM). *A priori* power analysis conducted using G*Power demonstrated that, at an alpha level of 0.05, with a medium effect size (*f*^2^ = 0.15) and up to five predictors, the study achieved a statistical power of 0.95, ensuring adequate sensitivity to detect meaningful effects. Correlational analyses revealed moderate to large effect sizes, with significant positive correlations between exposure to IPV and DERS (*r* = 0.48), between DERS and ED symptoms (*r* = 0.52), and between IPV and ED symptoms (*r* = 0.43). These psychometric properties and sample characteristics collectively support the methodological rigor and statistical robustness of the study. The full correlation matrix is provided in Supplementary Table S1.

### Additional analysis to assess potential attrition Bias

Given the inability to access data from participants who withdrew from the study due to ethical constraints, an indirect analysis was conducted to assess the potential risk of attrition bias within the retained sample. Specifically, participants were divided into two groups based on their response timing (early responders vs. late responders), with the latter considered more comparable to individuals at higher risk of dropout.

Independent samples t-tests revealed no statistically significant differences between early and late responders across key baseline variables, including IPV exposure, DERS total scores, and ED symptoms (all *p* > 0.05). Effect sizes were small, indicating minimal practical differences between the groups (see Supplementary Table S2).

These findings suggest that the final sample is unlikely to be substantially biased due to attrition, providing additional support for the robustness and generalizability of the study results.

### Results for hypotheses

To test hypotheses H1 to H3, Pearson correlation coefficients were calculated among the subdimensions of partner violence (economic control, economic exploitation, physical violence, emotional abuse, harassment), DERS (six subscales), and ED symptoms (dieting, bulimia and food preoccupation, oral control). The overall IPV composite score showed strong positive correlations with economic control (*r* = 0.85, *p* < 0.01), economic exploitation (*r* = 0.82, *p* < 0.01), psychological Abuse (*r* = 0.83, *p* < 0.01), physical violence (*r* = 0.78, *p* < 0.01), emotional abuse (*r* = 0.80, *p* < 0.01), and harassment (*r* = 0.70, *p* < 0.01), indicating that different forms of violence frequently co-occur. The total score for DERS was positively associated with overall IPV (*r* = 0.60, *p* < 0.01), with each of the six DERS subscales also showing moderate to strong correlations with IPV types (ranging from 0.30 to 0.55, *p* < 0.01), suggesting that exposure to IPV adversely affects multiple facets of emotional regulation, including nonacceptance of emotions, impulse control, and access to regulation strategies. High intercorrelations among the DERS subscales (ranging from 0.65 to 0.85, *p* < 0.01) further support the coherence of emotion regulation difficulties measured. Eating disorder symptoms, as measured by the EAT-26, correlated significantly with both total violence (*r* = 0.58, *p* < 0.01) and DERS scores (*r* = 0.65, *p* < 0.01). At the subscale level, dieting, bulimia and food preoccupation, and oral control showed moderate to strong correlations with violence types (*r* = 0.35 to 0.48) and DERS subscales (*r* = 0.50 to 0.60), highlighting the interplay between IPV, DERS, and ED across multiple symptom dimensions. These findings provide robust empirical evidence of the interconnectedness of IPV, DERS, and ED symptoms among married women in Jordan, warranting further multivariate analyses to explore causal pathways and mediation or moderation effects.

To examine hypotheses H4 and H5, longitudinal data collected at baseline and after 6 months from a sample of 308 married women exposed to IPV were analyzed using paired-sample t-tests and repeated measures ANOVA.

*H4*: Results indicated a statistically significant increase in DERS over the six-month period among women exposed to IPV. The total score on the DERS increased from a baseline mean of 72.4 (SD = 15.6) to 79.8 (SD = 17.2) at follow-up, t (307) = 8.21, *p* < 0.001, Cohen’s d = 0.53, reflecting a moderate effect size. Subscale analyses revealed significant increases across all six domains of the DERS, with the largest changes observed in Impulse Control Difficulties and Limited Access to Emotion Regulation Strategies (both *p* < 0.001). These findings corroborate the hypothesis that prolonged exposure to IPV exacerbates DERS over time.

*H5*: Similarly, the level of ED symptoms, also demonstrated a significant increase over 6 months among women who reported higher baseline DERS. The mean ED total score rose from 19.3 (SD = 8.7) at baseline to 24.5 (SD = 9.4) at follow-up, t (307) = 9.34, *p* < 0.001, Cohen’s d = 0.60, indicating a moderate to large effect size. The subscales of Dieting, Bulimia and Food Preoccupation, and Oral Control each exhibited significant upward trends (all *p* < 0.01). This pattern supports the proposition that worsening DERS contributes to escalating disordered eating behaviors longitudinally (See Table 1).

**Table 1:** Means and standard deviations of emotion regulation difficulties and eating disorder symptoms at baseline and follow-up.

Variable	Time point	*M*	SD
DERS	Baseline	72.4	15.6
	Follow-up	79.8	17.2
ED (EAT-26)	Baseline	19.3	8.7
	Follow-up	24.5	9.4

To test hypothesis H6, a longitudinal hierarchical regression analysis was conducted to examine whether changes in DERS (total score) over a six-month period predict corresponding changes in ED symptoms (EAT-26 total score) among women exposed to IPV (*N* = 308). Change scores were calculated by subtracting baseline scores from six-month follow-up scores for both variables. Results indicated that the change in DERS scores significantly predicted the change in EAT-26 scores, *β* = 0.58, t (306) = 12.03, *p* < 0.001, accounting for 33.6% of the variance in ED symptom changes (*R*^2^ = 0.336). This effect remained significant after controlling for baseline levels of both DERS and ED symptoms, as well as demographic covariates such as age and education. Further exploratory analyses of DERS subscales revealed that increases in Impulse Control Difficulties (*β* = 0.42, *p* < 0.001) and Limited Access to Emotion Regulation Strategies (*β* = 0.37, *p* < 0.001) were the strongest individual predictors of escalating ED symptoms.

A mediation analysis was performed using the PROCESS macro (Model 4) for SPSS with 5,000 bootstrap samples to test whether DERS (total score) mediates the relationship between exposure to IPV (composite violence score) and ED symptoms (EAT-26 total score) among women (*N* = 308) (H7). The total effect of IPV on ED symptoms was significant (c = 0.45, *p* < 0.001). When DERS was included as a mediator, the direct effect of IPV on ED symptoms decreased but remained significant (c’ = 0.21, *p* = 0.02), indicating partial mediation. The indirect effect through DERS was ab = 0.24, with a 95% bootstrap confidence interval of LLCI = 0.17 and ULCI = 0.32, demonstrating that the mediation effect is statistically significant, as the confidence interval does not include zero. This effect size indicates that difficulties in emotion regulation account for a substantial portion of the effect of IPV on ED symptoms, highlighting the crucial role of emotional regulation in this pathway.

These results suggest that exposure to IPV contributes to increased EDRS, which in turn leads to elevated symptoms of ED. EDRS account for a substantial portion of the effect of IPV on ED symptoms, highlighting the crucial role of emotional regulation processes in this pathway.

To examine H8, participants were divided into two groups: single-type violence (*n* = 130) and multiple-type violence (*n* = 178). In Table 2, an independent samples *t*-test showed that women in the multiple-type violence group had significantly higher scores on DERS (*M* = 3.74, SD = 0.52) compared to the single-type group (*M* = 3.10, SD = 0.47), *t* (306) = 10.23, *p* < 0.001, with a large effect size (Cohen’s d = 1.30). Similarly, the multiple-type violence group scored significantly higher on ED symptoms (*M* = 3.41, SD = 0.58) than the single-type violence group (*M* = 2.75, SD = 0.53), *t* (306) = 9.12, *p* < 0.001, Cohen’s d = 1.15.

**Table 2:** Means and standard deviations of DERS and ED symptoms by type of IPV.

Group	DERS (M ± SD)	ED Symptoms (M ± SD)
Single-type violence (*n* = 130)	3.10 ± 0.47	2.75 ± 0.53
Multiple-type violence (*n* = 178)	3.74 ± 0.52	3.41 ± 0.58

### Structural equation modeling analysis

#### Model specification and fit

**Figure 1:**
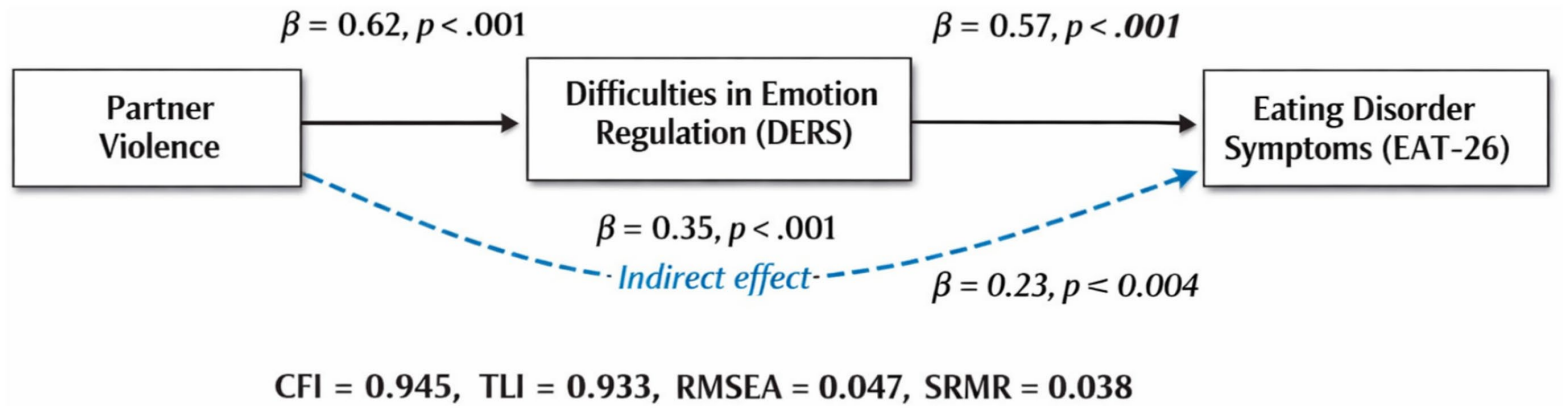
Structural equation modeling path diagram with standardized estimates (*β*) and *p*-values, and model fit indices.

A structural equation model was specified to examine the relationships between IPV exposure, DERS, and ED symptoms, including a mediation pathway whereby DERS mediate the effect of IPV on ED. The model was tested using IBM AMOS (Version 26). Measurement models for latent constructs (IPV, DERS, ED) were confirmed through confirmatory factor analysis (CFA) prior to testing the full structural model. The model demonstrated good fit to the data with the following indices: Comparative Fit Index (CFI) = 0.945, Tucker-Lewis Index (TLI) = 0.933, Root Mean Square Error of Approximation (RMSEA) = 0.047 (90% CI: 0.039–0.056), Standardized Root Mean Square Residual (SRMR) = 0.038. The model accounted for 48% of the variance in DERS and 54% of the variance in ED symptoms. These indices indicate an acceptable to excellent fit, supporting the adequacy of the model. The standardized path coefficients are summarized in Table 3, and Figure 1.

**Table 3:** Standardized path coefficients and hypothesis testing results.

Path	Standardized estimate (β)	SE	*p*-value
IPV → DERS	0.62	0.07	<0.001
DERS→ ED symptoms	0.57	0.06	<0.001
IPV → ED symptoms (Direct effect)	0.23	0.05	0.004
Indirect effect (IPV → DERS → ED)	0.35	—	<0.001

Table 3 shows that the strong positive path from IPV to DERS (*β* = 0.62) supports the hypothesis that exposure to multiple forms of IPV exacerbates DERS. The path from DERS to ED Symptoms (*β* = 0.57) confirms that greater DERS predicts higher severity of eating pathology. The direct path from IPV to ED Symptoms remains significant but is notably reduced compared to the total effect, indicating a partial mediation effect by DERS. Bootstrapped confidence intervals (5,000 samples) confirmed the significance of the indirect effect, reinforcing the mediating role of DERS.

## Discussion

To our knowledge, this study is among the first to examine the complex relationships between intimate partner violence (IPV), emotion dysregulation (EDRS), and eating disorders (ED). This investigation is particularly important because violence in intimate relationships is often recurrent and tends to escalate in severity over time. Our findings indicate that different forms of IPV frequently co-occur and are associated with greater difficulties in emotion regulation. Emotional abuse emerged as the most prevalent form, with impulse control difficulties representing the primary challenge in emotional dysregulation. Women experiencing higher levels of emotion regulation difficulties also exhibited more severe ED symptoms. Over time, exposure to partner violence exacerbated both EDRS and ED. Moreover, changes in EDRS predicted subsequent changes in ED symptoms and partially explained the pathway through which IPV influences these outcomes. Women exposed to multiple types of violence experienced more severe emotional and eating difficulties compared to those exposed to a single type.

While DERS and IPV explained approximately 54% of the variance in ED symptoms in the current study, nearly half of the variance remains unaccounted for. This unexplained variance highlights the potential contribution of additional psychological, social, and biological factors. Previous research has identified depression, post-traumatic stress disorder (PTSD), and low social support as significant predictors of disordered eating behaviors (Holmes et al., 2023; Cloitre et al., 2019). Other potential contributors include personality traits such as neuroticism, coping strategies, early-life trauma, and contextual factors such as socioeconomic status and cultural norms. Future studies should consider these variables to develop a more comprehensive understanding of the mechanisms underlying ED and to further clarify how IPV and emotion dysregulation influence eating behaviors. Accounting for these factors may also enhance predictive models and inform more targeted prevention and intervention strategies.

Although bidirectional relationships between IPV and eating disorders are theoretically possible, this study focused on a unidirectional pathway from IPV to ED. This approach aligns with prior empirical evidence showing that IPV often acts as a primary stressor, triggering maladaptive coping mechanisms such as disordered eating behaviors (Holmes et al., 2023; Huston et al., 2019). The current study design does not allow for the assessment of reverse causality, and therefore, the potential influence of pre-existing ED on subsequent IPV exposure could not be examined. Future longitudinal research is encouraged to explore possible bidirectional effects to provide a more complete understanding of the complex interplay between IPV and ED.

It is also important to note that, although the initial target sample size was 510 participants, only 308 completed the study due to personal circumstances such as divorce, emigration, and other challenges. Despite follow-up procedures—including phone calls, WhatsApp messages, and home visits—implemented to reduce attrition, the nearly 40% dropout rate may introduce selective bias. Participants who withdrew could differ systematically from those who completed the study in terms of IPV exposure, coping resources, or other demographic and psychological factors. This limitation should be considered when interpreting the generalizability and validity of the findings. Future studies are recommended to systematically compare completers and non-completers and implement additional strategies to minimize attrition and improve representativeness.

In addition to attrition-related concerns, it is important to consider the potential for survivorship bias in the current study. The final sample may disproportionately represent women who remained in abusive relationships, as participants who withdrew due to divorce, separation, or relocation were not retained in the analysis. Consequently, women who exited abusive relationships may be underrepresented. This limitation is particularly important because such women may differ systematically from those who remain, particularly in terms of emotional regulation capacities, coping strategies, and the severity of eating disorder symptoms. Therefore, the findings of this study may primarily reflect the experiences of women who continue to endure intimate partner violence, rather than the full spectrum of women exposed to such experiences. Accordingly, caution is warranted when generalizing the results to the broader population of women affected by IPV. Future research is encouraged to incorporate more diverse relationship trajectories and to examine whether psychological and behavioral patterns differ between women who remain in versus those who exit abusive relationships.

IPV remains a major global health and social issue, encompassing behaviors by a current or former intimate partner that cause physical, sexual, or psychological harm. These behaviors include physical aggression, sexual coercion, psychological abuse, controlling actions, as well as economic, emotional, verbal, and sexual abuse, in addition to control and humiliation, bullying, online abuse, harassment, and neglect-related violence. IPV manifests in multiple forms: ‘coercive controlling violence’ or ‘intimate terrorism’ involves one partner employing multiple strategies to dominate the other, whereas ‘situational couple violence’—the most frequent and generally less severe type—typically arises among younger couples (Alrawashdeh et al., 2024; Sa’Deh et al., 2024).

IPV is defined as any conduct within an intimate relationship that results in serious short- and long-term physical, mental, sexual, and reproductive health problems, imposing significant social and economic costs on women. Common psychological consequences of IPV include depression, post-traumatic stress disorder (PTSD), other anxiety disorders, sleep disturbances, eating disorders, and suicide attempts (World Health Organization, 2002; World Health Organization, 2012). Holmes et al. (2023) found that IPV exerts an indirect effect on ED through posttraumatic stress disorder (PTSD) symptoms. Specifically, IPV indirectly influenced weight and shape concerns, as well as binge eating symptoms via PTSD symptoms. While there was a significant total effect of IPV on compensatory behaviors, this effect was not mediated by PTSD symptoms.

The abuse of women constitutes a blatant violation of their fundamental rights and a serious form of gender-based discrimination. It can negatively affect all aspects of their lives and freedom of choice, under the dominance of deep-rooted cultures, traditions, and norms that support the patriarchal system. Multiple forms of abuse often intersect to exacerbate the harm inflicted on women, reflecting cultural constraints that limit their social status and roles, despite ongoing efforts to empower them (Alsawalqa R., 2021). Women in Jordan experience one or more forms of IPV from their husbands, ex-husbands, or romantic partners during their relationships. These forms include physical, verbal, emotional, psychological, economic, sexual, and cyber abuse. Women who faced economic abuse also endured primarily emotional and psychological abuse, followed by physical abuse and harassment, as tactics to reinforce economic abuse and maintain control over them (Alsawalqa et al., 2022; Alsawalqa R. O., 2021; Sa’Deh et al., 2024). The main factors driving this violence were identified as the patriarchal cultural context, gender roles, male dominance and coercive control, poverty, poor partner choice, financial gain, sexual exploitation, interference from family or peers, and incitement (Alsawalqa et al., 2022; Sa’Deh et al., 2024). Moreover, IPV—especially emotional abuse—can contribute to higher rates of marital burnout, primarily by increasing emotional exhaustion among employed married women. However, this exhaustion tends to decrease when women possess stronger emotional regulation skills, employing strategies such as cognitive reappraisal and expressive suppression (Tahat, 2024). A key aspect of burnout, including emotional exhaustion and a reduced sense of personal accomplishment are associated with eating disorder-related factors, such as emotional dissonance (Kristanto et al., 2016; Hage et al., 2021).

Eating disorders are serious mental health conditions characterized by abnormal eating behaviors and distorted attitudes toward food and body weight. These disorders often involve extreme emotions, attitudes, and behaviors surrounding weight and food, which can significantly impair physical health, psychological well-being, and social functioning. Common types include anorexia nervosa, bulimia nervosa, and binge-eating disorder. Eating disorder behavior has been defined as “dysfunctional attitudes toward eating as well as inadequate behavior toward food that is marked by anxiety, guilt, and fear, among many others factors” (Alvarenga Mdos et al., 2013). Diagnosis and classification of these disorders follow criteria outlined in the *Diagnostic and Statistical Manual of Mental Disorders* (5th ed.; American Psychiatric Association, 2013).

Eating attitudes encompass beliefs, thoughts, feelings, and behaviors related to food, and understanding them aids in explaining food choices and improving nutritional guidance. In eating disorder patients, these attitudes reveal clinical features and help predict eating behaviors, with their modification being crucial for successful treatment. Common attitudes in anorexia nervosa and bulimia nervosa include difficulty choosing food, categorizing food as good or bad, false nutrition beliefs, and using food to cope with emotions. These disorders share many of these traits, with some anorexia patients progressing to bulimia nervosa (Alvarenga Mdos et al., 2013).

Binge eating shows a stronger link to abuse than general overeating, with familial factors, IPV, and physical violence identified as key risk factors. Cumulative abuse experiences correlate specifically with binge eating behaviors rather than overeating (Bretaña et al., 2025). Moreover, females with obesity who have binge-eating disorder exhibit greater emotion dysregulation and emotional eating compared to those with obesity alone or food addiction (Ahmadkaraji et al., 2023). Notably, Women with lifelong eating disorders are at increased risk of experiencing physical and emotional intimate partner violence (IPV) during pregnancy and postpartum. This association is further influenced by the partner’s reaction to pregnancy and the mother’s history of childhood sexual abuse (Kothari et al., 2015).

Emotion regulation is more strongly related to IPV perpetration when modeled to predict an increased, rather than decreased, risk for IPV (Maloney et al., 2023). It also mediates the effects of violence victimization on nonsuicidal self-injury (Silva et al., 2017). Moreover, emotion regulation plays a significant mediating role in the relationship between exposure to abuse and psychological symptoms such as posttraumatic stress disorder and depression, with difficulties in emotion regulation influencing the severity of these symptoms (Cloitre et al., 2019). Similarly, emotion dysregulation mediates the relationship between the severity of borderline personality disorder symptoms and the progression of physical health symptoms over time (Gratz et al., 2017). Individuals exhibiting greater difficulties in emotion regulation are more likely to engage in health-risk behaviors such as self-harm, risky driving, violence, and unhealthy dietary habits compared to their peers (Singh and Singh, 2023). Furthermore, higher levels of DERS and separation anxiety are associated with more severe experiences of violence in marital relationships among women (Kahya and Taskale, 2019). In our findings, Impulse control difficulties were the most common among women, followed by non-acceptance of emotional responses and limited access to emotion regulation strategies, respectively. Difficulties engaging in goal-directed behavior and lack of emotional clarity were less common, while lack of emotional awareness was the least prevalent on average. This ranking indicates that challenges related to controlling emotional impulses represent the greatest obstacles in the emotion regulation process among women. Bucich and Maccann (2019) indicated that individuals’ self-beliefs about their emotional abilities, rather than emotional knowledge itself, influence the emotion regulation processes they use in daily life. Thus, women’s emotion regulation may often be an automatic (unconscious) process (Gross and Thompson, 2007).

On the other hand, women are particularly better at recognizing subtle emotions and are more sensitive to subtle cues of emotional expression, such as when the emotion is less intense or less typical. The emotional sensitivity hypothesis posits that women are more sensitive to subtle signals, meaning they perceive the intended emotion as more intense—but only when these signals are subtle or low in intensity. Men and women may not differ in recognizing clear and typical emotions, but women are more sensitive to emotional nuances and therefore have an advantage only in perceiving less intense or less typical emotional expressions. They may possess a more precise ability to identify emotions, especially sadness and surprise, as well as a faster detection of anger and disgust. Women often score higher than men on emotional intelligence or empathy tests, especially when measured through self-reports such as the Emotional Quotient Inventory, Empathy Quotient, Interpersonal Reactivity Index, or Emotional Awareness scales. They also outperform men in decoding emotional cues, particularly nonverbal ones. Nevertheless, there has been controversy in emotion studies regarding the stereotype that women are the more emotional sex and the belief that women are better at managing their own emotions and those of others. This stereotype influences self-perceptions; most research on emotional intelligence, personal sensitivity, or empathy measures shows that women consider themselves to be more emotionally intelligent, personally sensitive, and empathetic, raising another question posed by several studies: do these perceptions reflect their actual performance, or are they merely based on self-stereotypes? In this context, some studies have indicated that men focus more on subtle facial expressions and thus perceive more complex emotional profiles on the face—that is, men are better at perceiving emotional complexity. However, men tend to be less certain about their emotion recognition and become more easily confused when asked to rate the intensity of multiple emotions (Fischer et al., 2018; Tahat, 2024).

Women with higher levels of self-differentiation and more mature emotional defense mechanisms were less likely to experience addictive binge eating. Having strong psychological resources and mature emotional regulation mechanisms protects women from developing eating disorders, even when they are in relationships involving psychological abuse. Furthermore, these mechanisms acted as a *moderator* in the relationship between psychological abuse stress and addictive binge eating (Yona-Drori and Ben-Shlomo, 2021; Brewerton, 2007).

Overall, our findings emphasize the complex interplay between IPV and psychological functioning among married women, and they point to the critical importance of early interventions focused on enhancing emotional regulation to mitigate adverse health outcomes.

## Conclusion

This study reveals significant links between IPV, DERS, and ED symptoms among married women in Jordan. Exposure to various forms of IPV impairs emotion regulation—especially impulse control and access to effective strategies—worsening ED symptoms over time. EDRS partially mediate IPV’s effect on ED, with multiple violent types exacerbating these effects. These findings are situated within a patriarchal context that often tolerates male violence and sustains gendered power imbalances, intensifying women’s psychological burden.

There is a critical need for culturally sensitive interventions that focus on emotion regulation skills and psychological support, addressing both individual and structural factors. Specifically, Jordanian healthcare policymakers and domestic violence shelters should implement routine screening for eating disorders (ED) among women exposed to IPV. Staff training in recognizing and supporting emotional dysregulation, alongside integrated programs combining psychological counseling, emotion regulation training, and nutritional guidance, could significantly mitigate the adverse health outcomes of IPV.

Future research should further explore longitudinal mechanisms and develop tailored strategies to empower women affected by IPV, enhancing early detection, care pathways, and overall well-being in this vulnerable population.

## Data Availability

The original contributions presented in the study are included in the article/Supplementary material, further inquiries can be directed to the corresponding author/s.
