# Supplementary material for: Intimate partner violence and eating disorders among married women: the mediating role of emotion dysregulation
Source: Front Sociol. 2026 May 22;11:1747374. doi: 10.3389/fsoc.2026.1747374 (PMC13236538; doi:10.3389/fsoc.2026.1747374)
Supplement: Supplementary file 1 [file Data_Sheet_1.PDF]

Supplementary Table S1: Correlation Matrix Among IPV, DERS, and Eating Disorder Symptoms

Variable	IPV Total	Econ Control	Econ Exploitation	Emotional Abuse	Physical Abuse	Harassment	DERS Total	Nonacceptance	Impulse Control	Goal-directed	Awareness	Clarity	Strategies	ED Total	Dieting	Bulimia & Food Preoccupation	Oral Control
IPV Total	1	.85**	.82**	.80**	.78**	.70**	.48**	.38**	.40**	.36**	.35**	.36**	.37**	.43**	.40**	.42**	.43**
Econ Control	.85**	1	.75**	.70**	.65**	.60**	.38**	.32**	.34**	.30**	.30**	.31**	.32**	.36**	.34**	.35**	.36**
Econ Exploitation	.82**	.75**	1	.68**	.62**	.58**	.36**	.30**	.32**	.28**	.28**	.29**	.30**	.34**	.32**	.33**	.34**
Emotional Abuse	.80**	.70**	.68**	1	.66**	.55**	.40**	.33**	.36**	.32**	.32**	.33**	.34**	.38**	.36**	.37**	.38**
Physical Abuse	.78**	.65**	.62**	.66**	1	.50**	.35**	.30**	.32**	.28**	.28**	.29**	.30**	.32**	.30**	.32**	.32**
Harassment	.70**	.60**	.58**	.55**	.50**	1	.30**	.28**	.30**	.25**	.25**	.26**	.27**	.30**	.28**	.29**	.30**
DERS Total	.48**	.38**	.36**	.40**	.35**	.30**	1	.65**	.70**	.60**	.58**	.60**	.62**	.52**	.50**	.55**	.52**
Nonacceptance	.38**	.32**	.30**	.33**	.30**	.28**	.65**	1	.65**	.55**	.50**	.52**	.55**	.42**	.40**	.45**	.42**
Impulse Control	.40**	.34**	.32**	.36**	.32**	.30**	.70**	.65**	1	.60**	.58**	.60**	.62**	.50**	.48**	.52**	.50**
Goal-directed	.36**	.30**	.28**	.32**	.28**	.25**	.60**	.55**	.60**	1	.55**	.57**	.60**	.45**	.42**	.48**	.45**

Variable	IPV Total	Econ Control	Econ Exploitation	Emotional Abuse	Physical Abuse	Harassment	DERS Total	Nonacceptance	Impulse Control	Goal-directed	Awareness	Clarity	Strategies	ED Total	Dieting	Bulimia & Food Preoccupation	Oral Control
Awareness	.35**	.30**	.28**	.32**	.28**	.25**	.58**	.50**	.58**	.55**	1	.55**	.57**	.43**	.40**	.45**	.42**
Clarity	.36**	.31**	.29**	.33**	.29**	.26**	.60**	.52**	.60**	.57**	.55**	1	.60**	.45**	.42**	.48**	.45**
Strategies	.37**	.32**	.30**	.34**	.30**	.27**	.62**	.55**	.62**	.60**	.57**	.60**	1	.48**	.45**	.50**	.48**
ED Total	.43**	.36**	.34**	.38**	.32**	.30**	.52**	.42**	.50**	.45**	.43**	.45**	.48**	1	.50**	.55**	.52**
Dieting	.40**	.34**	.32**	.36**	.30**	.28**	.50**	.40**	.48**	.42**	.40**	.42**	.45**	.50**	1	.50**	.48**
Bulimia & Food Preoccupation	.42**	.35**	.33**	.37**	.32**	.29**	.55**	.45**	.52**	.48**	.45**	.48**	.50**	.55**	.50**	1	.50**
Oral Control	.43**	.36**	.34**	.38**	.32**	.30**	.52**	.42**	.50**	.45**	.42**	.45**	.48**	.52**	.48**	.50**	1

Note. r values represent Pearson correlation coefficients. ** $p < 0.01$

Supplementary Table S2: Comparison Between Early and Late Responders at Baseline Variables

Variable	Early Responders (n=154) M ± SD	Late Responders (n=154) M ± SD	t	p
IPV Total Score	2.78 ± 0.71	2.80 ± 0.72	0.32	0.75
DERS Total Score	72.3 ± 15.5	72.5 ± 15.6	0.18	0.86
ED Symptoms (EAT-26)	19.2 ± 8.6	19.4 ± 8.7	0.21	0.83
